# Phosphorus Is Associated with Coronary Artery Disease in Patients with Preserved Renal Function

**DOI:** 10.1371/journal.pone.0036883

**Published:** 2012-05-10

**Authors:** Ana Ludimila Cancela, Raul Dias Santos, Silvia Maria Titan, Patrícia Taschner Goldenstein, Carlos Eduardo Rochitte, Pedro Alves Lemos, Luciene Machado dos Reis, Fabiana Giorgetti Graciolli, Vanda Jorgetti, Rosa Maria Moysés

**Affiliations:** 1 Nephrology Department, University of São Paulo School of Medicine, São Paulo, Brazil; 2 Heart Institute, University of São Paulo School of Medicine Hospital das Clínicas, São Paulo, Brazil; University of Sao Paulo Medical School, Brazil

## Abstract

High serum phosphorus levels have been associated with mortality and cardiovascular events in patients with chronic kidney disease and in the general population. In addition, high phosphorus levels have been shown to induce vascular calcification and endothelial dysfunction *in vitro*. The aim of this study was to evaluate the relation of phosphorus and coronary calcification and atherosclerosis in the setting of normal renal function. This was a cross-sectional study involving 290 patients with suspected coronary artery disease and undergoing elective coronary angiography, with a creatinine clearance >60 ml/min/1.73 m^2^. Coronary artery obstruction was assessed by the Friesinger score and coronary artery calcification by multislice computed tomography. Serum phosphorus was higher in patients with an Agatston score >10 than in those with an Agatston score ≤10 (3.63±0.55 versus 3.49±0.52 mg/dl; *p* = 0.02). In the patients with Friesinger scores >4, serum phosphorus was higher (3.6±0.5 versus 3.5±0.6 mg/dl, *p* = 0.04) and median intact fibroblast growth factor 23 was lower (40.3 pg/ml versus 45.7 pg/ml, *p* = 0.01). Each 0.1-mg/dl higher serum phosphate was associated with a 7.4% higher odds of having a Friesinger score >4 (*p* = 0.03) and a 6.1% greater risk of having an Agatston score >10 (*p* = 0.01). Fibroblast growth factor 23 was a negative predictor of Friesinger score (*p* = 0.002). In conclusion, phosphorus is positively associated with coronary artery calcification and obstruction in patients with suspected coronary artery disease and preserved renal function.

## Introduction

High serum phosphorus has long been associated with increased mortality and cardiovascular events in patients with chronic kidney disease (CKD) [1], [2], and recent studies have extended those observations to the general population [3], [4]. In patients with preserved renal function, high-normal serum phosphorus has also been linked to the severity of coronary artery disease (CAD), as seen on coronary angiography [5], [6], as well as to increased carotid intima-media thickness [7] and ventricular hypertrophy [8].

There is increasing evidence that other mineral metabolism regulators are involved in the pathogenesis of cardiovascular disease. Phosphorus homeostasis is maintained by various hormones, mainly parathyroid hormone (PTH), calcitriol, and fibroblast growth factor 23 (FGF23). Excess PTH, as is seen in primary and secondary hyperparathyroidism [9], [10], is associated with increased mortality, and high PTH levels have been related to fatal events in the general population [11]. Vitamin D deficiency also has an impact on the incidence of cardiovascular events [12].

The phosphaturic hormone FGF23 decreases the production of 1,25-hydroxyvitamin D and PTH [13]. High phosphorus intake is the main stimulus for FGF23 synthesis by osteocytes [14]. In CKD patients, high levels of FGF23 have been associated with mortality [15], myocardial hypertrophy [16], and CAD [17]. Other populational studies have also shown that FGF23 is related to atherosclerosis [18], mortality, and cardiovascular events [19]. Those studies, however, included patients with renal dysfunction.

Therefore, in this study, we looked for associations between mineral metabolism and CAD in a cross-sectional study of patients with preserved kidney function and suspected CAD. We evaluated several markers of mineral homeostasis, as well as the presence of CAC and coronary artery obstruction, using the Agatston score (AS) to quantify CAC [20] and the Friesinger score (FS) to quantify coronary obstruction [21].

## Methods

### Participants

This was a cross-sectional study involving 290 clinically stable patients with suspected CAD submitted to elective coronary angiography, as ordered by their attending physicians, between June 2008 and December 2009 at the Heart Institute of *Hospital das Clínicas*, University of São Paulo School of Medicine. The inclusion criteria were an age higher than 18 years and an estimated Glomerular Filtration rate (eGFR)>60 ml/min/1.73 m^2^, as calculated by the Modification of Diet in Renal Disease formula [22]. Exclusion criteria were the presence of any hepatic, renal, autoimmune diseases, malignancy, myocardial revascularization, coronary angioplasty, and current use of bisphosphonates, calcium supplements, vitamin D supplements, steroids, or anticonvulsants. Data on medical history and anthropometry were obtained prior to coronary angiography, and blood sampling was drawn at the day of cardiac computed tomography.

**Table 1:** Basic characteristics of the study population^a^.

Characteristic	Value
Male, %	57.5
Age (years), mean ± SD	58.1±9.3
Race, %	
White	64.8
Non-white	35.2
Clinical data, %	
Hypertension	81.0
Diabetes	35.5
Dyslipidemia	45.2
Heart failure	19.7
Body mass index >25 kg/m^2^	28.6
Current smoking	32.1
Statin use	51.7
BMI (kg/m^2^), mean ± SD	28.0±4.9
SBP (mmHg), mean ± SD	131.2±18.8
DBP (mmHg), mean ± SD	82.3±11.9
Agatston score, median (IQR)	46 (0–297)
Friesinger score, median (IQR)	4 (4–9)
Biochemical data	
Alkaline phosphatase (U/L), mean ± SD	71±24
Total calcium (mg/dl), mean ± SD	9.5±0.5
Ionized calcium (mg/dl), median (IQR)	5.1 (5.0–5.3)
Phosphorus (mg/dl), mean ± SD	3.57±0.54
25(OH)D (ng/ml), mean ± SD	23.9±8.5
PTH (pg/ml), median (IQR)	57.5 (41–78.2)
FGF23 (pg/ml), median (IQR)	43.8 (27.0–68.8)
Creatinine (mg/dl), mean ± SD	0.89±0.17
Urea (mg/dl), median (IQR)	36.5 (30–44.3)
Glucose (mg/dl), median (IQR)	105 (94–121)
Apolipoprotein B (mg/dl), mean ± SD	86±25
Total cholesterol (mg/dl), mean ± SD	172±42
HDL cholesterol (mg/dl), median (IQR)	43 (36–52)
LDL cholesterol (mg/dl), mean ± SD	97±35
VLDL cholesterol (mg/dl), median (IQR)	26 (19–37)
Triglycerides (mg/dl), median (IQR)	131 (97–190)
C-reactive protein (mg/L), median (IQR)	2.8 (1.2–5.3)
C-peptide (ng/ml), median (IQR)	2.6 (1.8–3.7)
eGFR (ml/min/1.73 m^2^), median (IQR)	92.0 (79.1–109.0)

BMI, body mass index; SBP, systolic blood pressure; DBP, diastolic blood pressure; 25(OH)D, 25-hydroxyvitamin D; HDL, High-density lipoprotein; LDL, Low-density lipoprotein; VLDL, very low-density lipoprotein; eGFR, estimated GFR.

a Normally distributed variables are expressed as mean ± SD and non-normally distributed variables are expressed as median (IQR).

**Table 2:** Characteristics of the patients evaluated, by Agatston score^a^.

Characteristic	No-CAC Group	CAC Group	*p*
	AS≤10	AS>10	
	(n = 121)	(n = 169)	
Age (years), mean ± SD	54.7±8.8	60.4±8.9	<0.001
Male gender, %	49.3	63.3	0.02
Hypertension, %	72.7	86.9	0.004
Diabetes, %	24.8	43.2	0.001
Current smoking, %	29.7	33.7	0.5
Statin use, %	42.9	57.9	0.01
Alkaline phosphatase (U/L), mean ± SD	76±22	78±25	0.89
Total calcium (mg/dl), mean ± SD	9.4±0.5	9.5±0.5	0.2
Ionized calcium (mg/dl), median (IQR)	5.1 (4.9–5.3)	5.1 (5.0–5.3)	0.1
Phosphorus (mg/dl), mean ± SD	3.5±0.5	3.6±0.5	0.02
25(OH)D (ng/ml), mean ± SD	23.6±8.4	24.2±8.7	0.43
PTH (pg/ml), median (IQR)	61 (43.5–85)	53 (38–76)	0.06
FGF23 (pg/ml), median (IQR)	45.7 (28.6–76.8)	42.1 (26.1–65.3)	0.21
Creatinine (mg/dl), mean ± SD	0.9±0.2	0.9±0.2	0.9
Urea (mg/dl), median (IQR)	37 (29–45)	36 (30–44)	0.8
Glucose (mg/dl), median (IQR)	101 (91.5–119)	106 (96–129)	0.03
Apolipoprotein B (mg/dl), mean ± SD	92±28	82±22	0.006
Total cholesterol (mg/dl), mean ± SD	182±45	164±37	<0.001
HDL cholesterol (mg/dl), median (IQR)	44 (35–54)	43 (36–52)	0.8
LDL cholesterol (mg/dl), mean ± SD	106±37	91±31	<0.001
VLDL cholesterol (mg/dl), median (IQR)	26 (10–405)	26 (20–36)	0.6
Triglycerides (mg/dl), median (IQR)	132 (94–202)	129 (99–181)	0.5
C-reactive protein (mg/L), median (IQR)	2.7 (1.3–4.9)	2.9 (1.2–5.7)	0.6
C-peptide (ng/ml), median (IQR)	2.6 (1.8–3.5)	2.6 (1.8–3.7)	0.5
eGFR (ml/min/1.73 m^2^), median (IQR)	90.5 (77.6–106.6)	93.1 (79.6–110.7)	0.5
Friesinger score, median (IQR)	0 (0–3)	8 (4–11)	<0.001

25(OH)D, 25-hydroxyvitamin D; PTH, parathyroid hormone; HDL, High-density lipoprotein; LDL, Low-density lipoprotein, VLDL, very low-density lipoprotein, eGFR, estimated GFR.

a Normally distributed variables are expressed as mean ± SD and non-normally distributed variables are expressed as median (IQR).

**Figure 1:**
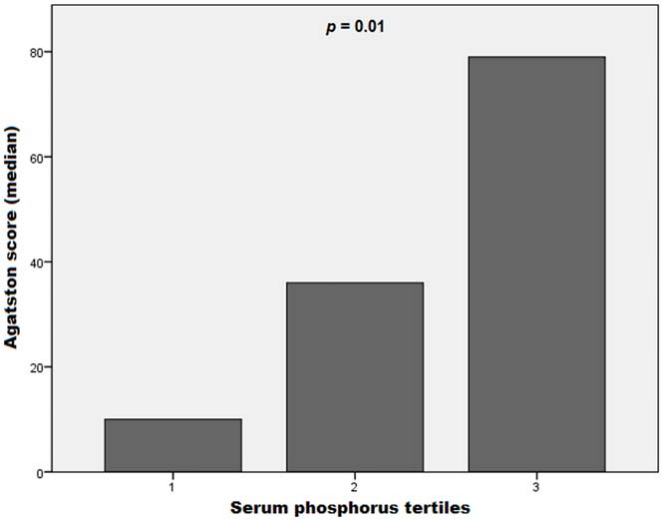
Agatston scores by serum phosphorus tertiles. Results are expressed as medians. Agatston scores were progressively higher across phosphorus tertiles (*P* = 0.01).

**Table 3:** Univariate and multivariate logistic regression analysis of the risk of having an Agatston score >10.

Variable	OR	95% CI	*p*
***Univariate analysis***			
Age	1.07	1.04–1.10	<0.001
Male gender	1.75	1.09–2.82	0.02
White	1.32	0.81–2.14	0.27
Diabetes	2.31	1.38–3.85	0.001
Hypertension	2.51	1.37–4.57	0.003
Smoking	0.83	0.51–1.37	0.47
Phosphorus	1.61	1.03–2.51	0.03
Total calcium	1.42	0.86–2.33	0.17
Alkaline phosphatase	1.00	0.99–1.01	0.68
Glucose	1.00	0.99–1.01	0.19
LDL cholesterol	0.99	0.98–0.99	<0.001
eGFR (ml/min/1.73 m^2^)	1.00	0.99–1.01	0.74
Log PTH	0.34	0.11–0.99	0.05
Log FGF23	0.56	0.26–1.22	0.14
***Multivariate models***			
***Model 1***			
Phosphorus	1.56	0.99–2.14	0.05
Log FGF23	0.58	0.26–1.27	0.17
Log PTH	0.39	0.13–1.17	0.09
***Model 2***			
Phosphorus	1.98	1.19–3.29	0.01
Age	1.08	1.05–1.11	<0.001
Male gender	3.02	1.72–5.28	<0.001
Hypertension	2.32	1.18–4.54	0.01
Diabetes	1.82	1.04–3.18	0.04
***Model 3***			
Phosphorus	1.92	1.56–3.19	0.01
Age	1.08	1.05–1.12	<0.001
Male gender	2.85	1.62–5.02	<0.001
Hypertension	2.36	1.19–4.66	0.01
Diabetes	1.77	1.01–3.13	0.05
Log PTH	0.42	0.12–1.45	0.17
Log FGF23	0.55	0.23–1.31	0.18

**Table 4:** Characteristics of the patients evaluated, by Friesinger score^a^.

Characteristic	Friesinger score	Friesinger score	*p*
	≤4	>4	
	(n = 149)	(n = 137)	
Age (years), mean ± SD	55.8±9.2	60.3±8.9	<0.001
Male gender, %	76 (51.0)	89 (64.9)	0.02
Hypertension, %	111 (74.5)	122 (89.05)	0.002
Diabetes, %	43 (38.8)	59 (43.0)	0.01
Current smoking, %	46 (30.8)	45 (32.8)	0.72
Statin use, %	59 (39.6)	89 (64.9)	<0.001
Alkaline phosphatase (U/L), mean ± SD	75.3±23.6	79.5±24.6	0.06
Total calcium (mg/dl), mean ± SD	9.4±0.5	9.5±0.5	0.80
Ionized calcium (mg/dl), median (IQR)	5.1 (4.9–5.3)	5.1 (5.0–5.3)	0.27
Phosphorus (mg/dl), mean ± SD	3.5±0.6	3.6±0.5	0.04
25(OH)D (ng/ml), mean ± SD	23.8±8.6	24.2±8.4	0.42
PTH (pg/ml), median (IQR)	60 (43–79)	52 (36–79)	0.18
FGF23 (pg/ml), median (IQR)	45.7 (31.7–76.1)	40.3 (24.1–62.2)	0.01
Creatinine (mg/dl), mean ± SD	0.87±0.16	0.90±0.18	0.14
Urea (mg/dl), median (IQR)	36 (29–44)	37 (30–44)	0.63
Glucose (mg/dl), median (IQR)	104 (92–121)	105 (95–122)	0.33
Apolipoprotein B (mg/dl), mean ± SD	89±26	84±23	0.35
Total cholesterol (mg/dl), mean ± SD	177±43	165±40	0.03
HDL cholesterol (mg/dl), median (IQR)	45 (36–54)	42 (35–50)	0.05
LDL cholesterol (mg/dl), mean ± SD	101±36	93±32	0.08
VLDL cholesterol (mg/dl), median (IQR)	25 (18–39)	26 (20–36)	0.52
Triglycerides (mg/dl), median (IQR)	129 (92–196)	131 (101–185)	0.56
C-reactive protein (mg/L), median (IQR)	2.5 (1.3–4.5)	3.2 (1.2–6.2)	0.14
C-peptide (ng/ml), median (IQR)	2.7 (1.9–3.6)	2.6 (1.8–3.7)	0.86
eGFR (ml/min/1.73 m^2^), median (IQR)	93.7 (79.7–106.7)	92.3 (79.4–11.2)	0.89
Agatston score	0 (0–35)	214 (87–838)	<0.001

25(OH)D, 25-hydroxyvitamin D; PTH, parathyroid hormone; HDL, High-density lipoprotein; LDL, Low-density lipoprotein, VLDL, very low-density lipoprotein, eGFR, estimated GFR.

a Normally distributed variables are expressed as mean ± SD and non-normally distributed variables are expressed as median (IQR).

**Table 5:** Univariate and multivariate logistic regression of the risk of having a Friesinger score higher than the median (4 points).

Variable	OR	95% CI	*p*
***Univariate analysis***			
Age	1.06	1.03–1.08	<0.001
Male gender	1.78	1.11–2.87	0.02
White	1.96	1.19–3.22	0.01
Diabetes	1.86	1.14–3.04	0.01
Hypertension	2.78	1.45–5.34	0.002
Phosphorus	1.47	0.95–2.28	0.08
Agatston score (increase of 10)	1.06	1.03–1.07	<0.001
Log PTH	0.47	0.16–1.35	0.16
Log FGF23	0.32	0.14–0.71	0.005
***Multivariate models***			
***Model 1***			
Phosphorus	1.59	1.02–2.48	0.04
Male gender	1.90	1.17–3.09	0.01
***Model 2***			
Phosphorus	1.73	1.08–2.76	0.02
Male gender	2.43	1.44–4.09	0.001
Age	1.07	1.04–1.09	<0.001
***Model 3***			
Phosphorus	1.75	1.05–2.81	0.02
Male gender	2.63	1.54–4.47	<0.001
Age	1.06	1.03–1.09	<0.001
White	1.95	1.14–3.31	0.01
***Model 4***			
Phosphorus	1.76	1.08–2.86	0.02
Male gender	2.61	1.51–4.51	<0.001
Age	1.07	1.03–1.09	<0.001
White	2.02	1.17–3.49	0.01
Log PTH	0.72	0.23–2.31	0.59
Log FGF23	0.28	0.12–0.67	0.003
***Model 5***			
Phosphorus	1.73	1.06–2.82	0.03
Male gender	2.94	1.68–5.09	<0.001
Age	1.06	1.03–1.09	<0.001
White	1.99	1.16–3.41	0.01
Hypertension	2.71	1.34–5.49	0.01
Diabetes	1.46	0.85–2.49	0.16
***Model 6***			
Phosphorus	1.74	1.06–2.88	0.03
Male gender	2.97	1.69–5.23	<0.001
Age	1.06	1.03–1.09	<0.001
White	2.07	1.19–3.61	0.01
Hypertension	2.79	1.36–5.70	0.01
Diabetes	1.51	0.87–2.61	0.15
Log PTH	0.77	0.23–2.54	0.67
Log FGF23	0.26	0.11–0.63	0.002

### Ethics Statement

The study was reviewed and approved by the local institutional ethics board, *Comitê de Ética para a Análise de Projetos de Pesquisa do Hospital das Clínicas da Faculdade de Medicina da Universidade de São Paulo* (process number 0755/07). All participating patients gave written informed consent.

### Biochemical Analysis

Whole blood was collected after a 12 h fast, and creatinine, urea, total calcium, ionized calcium, phosphorus, intact PTH (immunochemiluminometric assay, reference range, 16–87 pg/ml; DPC, Los Angeles, California), total alkaline phosphatase, 25(OH)D (radioimmunoassay kit, reference range >30.0 ng/ml; DiaSorin, Stillwater, Minnesota), intact FGF23 (ELISA kit, reference range 27.8–50.0 pg/ml; Kainos, Tokyo, Japan), C-reactive protein, total cholesterol, high- and low-density lipoprotein cholesterol, triglycerides, apolipoprotein B, glucose, and C-peptide were determined.

### Cardiac Computed Tomography

Multislice computed tomography was performed in a scanner with 64 detector rows (Aquilion 64; Toshiba, Otawara, Japan). To determine the initial and final level of the scan, all patients were monitored electrocardiographically for synchronization with image acquisition. A chest X-ray was acquired in apnea. Images were obtained using a protocol in which 64 slices (each 3 mm thick) were obtained, with image acquisition triggered at 80% of the RR interval. Calcification was quantified by calculating the AS on a workstation (Vitrea 2, version 3.5; Vital Images Inc, Plymouth, Minnesota). Coronary calcifications were defined as four or more contiguous pixels with a density of ≥130 Hounsfield units. The total AS was calculated as the sum of the individual scores for the four major epicardial coronary arteries [20].

### Coronary Angiography

Patients were submitted to coronary angiography in the Hemodynamics Laboratory of the Heart Institute, and standard procedures were used. To evaluate the burden of atherosclerotic disease, Friesinger score was calculated. In order to calculate the FS, left anterior descendent, circumflex, and right coronary arteries each receive 0–5 points depending on the degree of coronary obstruction, and the total FS being the sum of those three values) [21].

### Statistical Analysis

Continuous parametric variables are expressed as mean ± standard deviation, and nonparametric variables are expressed as median and interquartile range. For the univariate analysis, we used the Mann-Whitney and the chi-square tests. Kruskal-Wallis test was applied for the analyses of tertiles of phosphorus.

We then constructed several univariate and multivariate linear regression models using phosphorus and log FGF23 as the dependent variables. For the logistic regression analyses, our patients were divided according to CAC (no-CAC group, AS≤10; and CAC group, AS>10) [23] and Friesinger values (higher and upper the median value).

All tests were two-tailed, and the level of significance was set at 5%. All statistical analyses were performed with the Statistical Package for the Social Sciences, version 13.0 (SPSS Inc., Chicago, Illinois).

## Results

### Baseline Clinical and Laboratory Characteristics

Table 1 shows the baseline characteristics of the study population, with a significant proportion of diabetics and hypertensive (35.5 and 81.0%, respectively). The median AS was 46.5. We found the prevalence of hypovitaminosis D (<30 ng/ml) to be 75.9% in the study population.

### Calcification

Of the 290 patients evaluated, 92 (31.7%) had an AS of 0. When patients were classified according to AS (Table 2), those with an AS>10 (CAC group) were older and showed a higher frequency of male gender, hypertension, and diabetes. In the CAC group, serum phosphorus was significantly higher and PTH showed a near-significant association with CAC (p = 0.06). There were no differences between the two groups regarding FGF23, ionized calcium, alkaline phosphatase, or 25(OH)D. In addition, the CAC group presented lower levels of apolipoprotein B, total cholesterol, and low-density lipoprotein cholesterol, and a higher frequency of statins use.

When the population was divided into tertiles of serum phosphorus, we observed that median AS increased progressively (*p* = 0.01), as shown in Figure 1.

Table 3 shows the logistic regression related to the risk of having an AS>10. In the univariate analysis, we found that each 0.1-mg/dl rise in serum phosphorus was associated with a 6.1% higher odds of having an AS>10. Phosphorus remained a significant risk factor for CAC even after adjustment for multiple variables such as age, gender, diabetes, hypertension, FGF23 level, and PTH concentration. As expected, traditional risk factors such as age, gender, hypertension, and diabetes were also strong predictors of an AS>10. In the univariate analysis, log PTH was negatively associated with the risk of CAC, although this association disappeared in the multivariate analysis (Table 3). Other mineral metabolism markers, including FGF23, calcium, and alkaline phosphatase, were not predictors of CAC in our sample.

### Coronary Angiography

Table 4 shows the characteristics of the patients by FS. Mean age was higher in the >4 FS group than in the ≤4 FS group, as were the proportions of males, hypertensive patients, diabetic patients, and patients using statins. Serum phosphorus was significantly higher and FGF23 was lower in the group with higher Friesinger score.

Table 5 shows the logistic regression models on Friesinger score. Age, male gender, hypertension, diabetes and AS were all significantly associated with FS, whereas, in the univariate analysis, serum phosphorus was not. However, after adjustment for gender and other variables (gender, race, age, hypertension, diabetes, logPTH and logFGF23), serum phosphorus showed a positive association, with 0.1-mg/dl increase in phosphorus being associated with a 7.4% higher odds of having higher Friesinger score. Conversely, FGF23 was a negative predictor of FS, both in the univariate and multivariate models.

### Biochemical Variables

We attempted to identify the determinants of serum phosphorus and FGF23. Serum phosphorus was higher in females than in males (3.65±0.49 mg/dl versus 3.52±0.57 mg/dl; *p* = 0.04), a fact in accordance to the effect of gender in the logistic regression models. Interestingly, serum phosphorus did not correlate significantly with other mineral metabolism markers, such as FGF23, PTH, 25(OH)D, and calcium. In the stepwise linear regression model including phosphorus as the dependent variable and age, sex, race, smoking status, hypertension, diabetes, BMI, 25(OH)D, logPTH, logFGF23, urea and creatinine clearance as possible determinants, only urea, creatinine clearance, and gender remained as determinants of serum phosphorus, collectively explaining 6% of its variation (R^2^ = 0.06).

We found that FGF23 correlated positively with total calcium (R = 0.197; *p*<0.001) but not with serum phosphorus, logPTH, or 25(OH)D. We also performed a stepwise linear regression model on the FGF23 adjusted for age, gender, race, smoking, hypertension, diabetes, body mass index, 25(OH)D, total calcium, phosphorus, log PTH, urea, and creatinine clearance. Again, only total calcium remained significantly associated with serum intact FGF23, explaining 3% of the variation in the latter (R^2^ = 0.03).

## Discussion

To our knowledge, this is the first study to evaluate the associations between CAD and all of the major mineral metabolism markers, including phosphorus, FGF23, 25(OH)D, PTH, and calcium. It was also the first study to evaluate such associations using CAC and coronary artery obstruction as outcome measures in patients with preserved kidney function. Even after adjusting for traditional cardiovascular risk factors, we found that serum phosphorus was a strong predictor of CAC and coronary artery obstruction, whereas FGF23 correlated negatively with such obstruction.

The association between serum phosphorus and cardiovascular risk was first demonstrated in the CKD population. It has also been associated with mortality [1], [2] and vascular calcification in CKD [24]. Subsequent studies [3], [4] showed, for the first time, that high-normal serum phosphorus levels are also associated with mortality and cardiovascular events in the general population and there is increasing evidence that phosphorus is associated with atherosclerosis and CAD. It has been shown that phosphorus is an independent predictor of carotid intima-media thickness in the general population [7]. In an earlier study, Narang *et al.* demonstrated a link between phosphorus and coronary artery obstruction in the absence of renal dysfunction [5]. The authors studied more than 300 patients and found that each 0.5-mmol/L increase in serum phosphorus resulted in a 3 times greater chance of having significant coronary disease [5]. Two longitudinal studies recently found serum phosphorus to be a marker of incident and progressive CAC in low-risk patients [25], [26]. Our findings confirm the predictive value of serum phosphorus in high-risk patients. Likewise, we found that for each 0.1-mg/dl increase in serum phosphorus the odds of having an abnormally high FS increased by 7.4%.

*In vitro* studies have shown that hyperphosphatemia induces a phenotypic transformation of vascular smooth muscle cells into osteoblast-like cells that express biochemical markers characteristic of the bone lineage, such as Runx2 [27], leading to calcification [27]. In addition, high dietary phosphorus intake has been shown to cause endothelial dysfunction in young men [28], and it has been demonstrated that a phosphorus-enriched medium induces bovine aortic endothelial cells to produce greater quantities of reactive oxygen species and less nitric oxide [28]. Endothelial dysfunction and vascular calcification are both involved in the atherosclerotic process, and obstructive atherosclerotic lesions are closely linked to vascular wall calcifications [29]. Vascular calcification increases the risk of cardiovascular events, and an AS>10 significantly increases mortality from cardiovascular disease [23].

In CKD patients, FGF23 is associated with CAC [30], coronary obstruction [17], and mortality [15]. A recent *post hoc* analysis of the Heart and Soul study found FGF23 to be a predictor of mortality [19] in the general population. In addition, analyses derived from the Prospective Investigation of the Vasculature in Uppsala Seniors (PIVUS) study also demonstrated a positive relationship between FGF23 and total body atherosclerosis in a sample of 70-year-old patients [18]. These results are not in accordance with our findings, which showed an inverse association between FGF23 and coronary obstruction and no association between FGF23 and CAC, a result in agreement with a previous study [31]. One major explanation for that is that renal dysfunction was not an exclusion criteria in the PIVUS and the positive relationship between FGF23 and atherosclerosis might result from that, particularly in older patients. Another possibility is that the inverse association of FGF23, which persisted even after adjustment for serum phosphorus itself, might reflect in patients with normal kidney function the burden of phosphorus load and the balance between FGF23 and all other mineral metabolism elements.

In the absence of renal dysfunction, however, FGF23 deficiency is characterized by hyperphosphatemia and ectopic calcifications [32]. In contrast, excessive FGF23 production or activity results in hypophosphatemia, osteomalacia [33], and rickets [34]. Nevertheless, there have been no reports of accelerated atherosclerotic disease or increased cardiovascular events in individuals with preserved renal function and high FGF23 levels, probably because they are frequently hypophosphatemic. Another relevant difference between populations with and without renal dysfunction is that the expression of klotho, a cofactor for FGF receptors activation by FGF23 [35], is significantly impaired in the first group. Therefore, the analysis of the associations of FGF23 with coronary disease should be done separately from those patients with CKD, even with a minor impairment, since the decrease of Klotho expression is an early event in CKD [36].

This study has some limitations. First, the cross-sectional design precludes the evaluation of causality and temporality among the variables. Second, CAC and coronary obstruction are intermediary outcomes, and, despite being good markers of coronary events, the associations found here cannot be extrapolated to clinical outcomes such as mortality and cardiovascular events. Third, calcitriol levels, phosphate excretion, and dietary intake were not assessed, which could have improved the understanding of phosphorus homeostasis in this population. Finally, the differences in serum phosphorus between both the Agatston Score and Friesinger Score groups were small (3.6 vs 3.5 mg/dl) and, although they were statistically significant, this could mean a type A error. Nevertheless, our patient sample was relatively large, homogeneous and did not include patients with renal dysfunction. We also excluded patients using medications or supplements that might affect mineral and bone metabolism. Various mineral metabolism markers were measured simultaneously, and CAD was evaluated by quantifying CAC and obstruction in the same set of patients. There have been no previous cross-sectional studies demonstrating the value of phosphorus as a predictor of CAC.

Serum phosphorus is considered a nontraditional cardiovascular risk factor in the CKD population and might confer risk in the general population as well. We increasingly consume less natural food and more processed food. Food processing typically introduces high-phosphate additives and might be partially responsible for phosphate intakes being well above the recommended levels [37] and the deleterious effects of a high-phosphate diet have yet to be fully understood. Considering the increasing evidence of an association between phosphorus and cardiovascular disease, it is important to prospectively investigate whether interventions aimed at reducing the phosphate burden, such as restricting dietary phosphorus and limiting the use of food additives, reduce cardiovascular events and mortality in high-risk patients or in the general population. The relationship between FGF23 and cardiovascular disease in the absence of CKD also merits further investigation.

In conclusion, we found that, in a clinically stable population with suspected CAD and preserved renal function, serum phosphorus was strongly and consistently associated with CAC and coronary obstruction, as well as that FGF23 was negatively associated with coronary obstruction.
